# 585 A Quality Improvement Project to Impact Adult and Pediatric No-Show Rates

**DOI:** 10.1093/jbcr/irae036.219

**Published:** 2024-04-17

**Authors:** Peggy Barmore, Emily Webb, Kati Venable, Ashley Burnette, Jane D Echols, Samantha Schech, Richard Cartie, Rajiv Sood

**Affiliations:** Doctors Hospital of Augusta, Augusta, GA; JMS Burn Centers, Augusta, GA; JMS Burn Center at Doctors Hospital Augusta, Augusta, GA; Doctors Hospital of Augusta, Augusta, GA; JMS Burn Centers, Augusta, GA; JMS Burn Center at Doctors Hospital Augusta, Augusta, GA; Doctors Hospital of Augusta, Augusta, GA; JMS Burn Centers, Augusta, GA; JMS Burn Center at Doctors Hospital Augusta, Augusta, GA; Doctors Hospital of Augusta, Augusta, GA; JMS Burn Centers, Augusta, GA; JMS Burn Center at Doctors Hospital Augusta, Augusta, GA; Doctors Hospital of Augusta, Augusta, GA; JMS Burn Centers, Augusta, GA; JMS Burn Center at Doctors Hospital Augusta, Augusta, GA; Doctors Hospital of Augusta, Augusta, GA; JMS Burn Centers, Augusta, GA; JMS Burn Center at Doctors Hospital Augusta, Augusta, GA; Doctors Hospital of Augusta, Augusta, GA; JMS Burn Centers, Augusta, GA; JMS Burn Center at Doctors Hospital Augusta, Augusta, GA; Doctors Hospital of Augusta, Augusta, GA; JMS Burn Centers, Augusta, GA; JMS Burn Center at Doctors Hospital Augusta, Augusta, GA

## Abstract

**Introduction:**

The literature shows that no-show appointments result in lost revenue and impact key outcomes for patients, such as decreased access, leading to patient dissatisfaction and decreased provider productivity. In trying to determine a national benchmark for no-show rates in a wound and burn clinic, not much information is forthcoming. No-show rates ranged from 19% to as high as 49%. Lost revenue from no-shows was shown to range from $191k-$384k per year. The goal of this quality improvement project is to share our process of a personal call-back protocol based on a risk stratification that resulted in improved pediatric no-show rates.

**Methods:**

Our environment is a high-volume advanced wound and burn care for adults and pediatrics, with just over 35,000 visits annually. It was noted that monitoring follow-up care and missed appointments is essential in providing high-quality wound and burn care. An undetermined number of patients were not returning for follow-up appointments. In 2022, we began to monitor no-show trends with a goal of monitoring for 6 months to determine what our goal rate should be. We separated adults from pediatrics and measured a no-show as a percentage of patients who did not attend their appointments, compared to all patients given appointments. A personal call-back procedure was implemented to contact patients or parents and determine the reason for not returning and to offer a reminder or reschedule for the next appointment. In six months, we set our adult no-show goal at 5% and our pediatric no-show goal at 10%. Various measurement sources were generated to report outcomes:

• Daily no-show report reviewed weekly at a minimum

• Monthly no-show rate compiled and reported to Burn Quality and Safety Committee

• Stratification of importance of follow-up with support from providers for pediatric patients. (High Risk, Medium Risk, Low Risk).

Additionally, we sought support and help from our local burn foundation to assist with transportation issues.

**Results:**

A pediatric no-show rate of 15.75% was decreased to 9.41% in one year and the adult no-show rate was decreased from 1.44% to 0.32% in one year.

**Conclusions:**

A live, personal call procedure for adult and pediatric patients results in decreased no-show rates in an advanced wound and burn clinic. Stratification of patient risk for the pediatric population helps target those most likely not to return. Garnering support from our local burn foundation helped meet a gap to overcome the barriers patients were encountering.

**Applicability of Research to Practice:**

Lowering no-show rates not only helps with maintaining a thriving outpatient practice, but ensures that patients continue to receive consistent care after the transition from acute to long-term care. Furthermore, the stratification and identification of patients that are at a higher risk of not returning allows for more personalized attention from the clinic staff in order to meet those patients' needs.

